# Recent Progress in Enzyme Immobilization to Metal–Organic Frameworks to Enhance the CO_2_ Conversion Efficiency

**DOI:** 10.3390/molecules30020251

**Published:** 2025-01-10

**Authors:** Yunhan Cao, Pengyan Yang, Rui Zhao, Fenghuan Wang

**Affiliations:** School of Light Industry, Beijing Technology and Business University (BTBU), Beijing 100048, China; caoyunhan1119@163.com (Y.C.);

**Keywords:** CO_2_ conversion, enzyme@MOF, coenzyme regeneration, multi-enzyme co-immobilization

## Abstract

Climate change and the energy crisis, driven by excessive CO_2_ emissions, have emerged as pressing global challenges. The conversion of CO_2_ into high-value chemicals not only mitigates atmospheric CO_2_ levels but also optimizes carbon resource utilization. Enzyme-catalyzed carbon technology offers a green and efficient approach to CO_2_ conversion. However, free enzymes are prone to inactivation and denaturation under reaction conditions, which limit their practical applications. Metal–organic frameworks (MOFs) serve as effective carriers for enzyme immobilization, offering porous crystalline structures that enhance enzyme stability. Moreover, their high specific surface area facilitates strong gas adsorption, making enzyme@MOF composites particularly advantageous for CO_2_ catalytic conversion. In this paper, we review the synthesis technologies and the application of enzyme@MOFs in CO_2_ catalytic conversion. Furthermore, the strategies, including the enhancement of CO_2_ utilization, coenzyme regeneration efficiency, and substrate mass transfer efficiency, are also discussed to further improve the efficiency of enzyme@MOFs in CO_2_ conversion. The aim of this review is to present innovative ideas for future research and to highlight the potential applications of enzyme@MOFs in achieving efficient CO_2_ conversion.

## 1. Introduction

**Figure 1:**
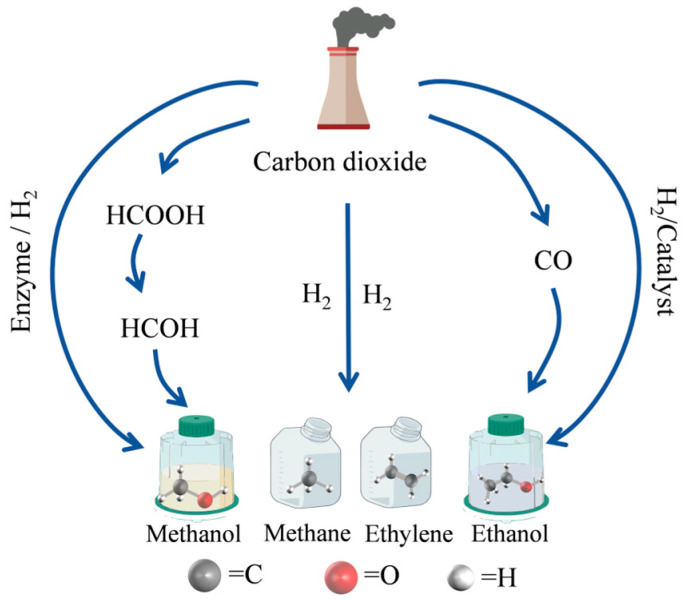
Schematic diagram of CO_2_ conversion pathway.

With the ongoing increase in the world population and the improvement in living quality, there is a corresponding increase in the need for energy. Consequently, there is a continuous increase in carbon dioxide (CO_2_) emissions on an annual basis. According to the China Greenhouse Gas Bulletin 2023, the global atmospheric CO_2_ concentration reached 417.9 ppm in 2022, the highest level in the past two million years. The data indicate that anthropogenic CO_2_ emissions have persistently risen and have surpassed the acceptable limit for the CO_2_ concentration in the atmosphere [1]. The widespread emission of CO_2_ has become a critical global concern that necessitates immediate attention. The sustainability of economic growth, ecosystems, and human lives may be substantially impacted by the consequences of climate change and the occurrence of extreme weather events triggered by excess CO_2_ emissions [2,3,4]. Consequently, the international community has been increasingly focused on investigating efficient strategies to convert and utilize CO_2_ in order to attain global carbon neutrality (Figure 1).

A mutually beneficial solution for the efficient utilization of CO_2_ is to capture and transform it into high-value goods, which can successfully lower atmospheric concentrations of carbon dioxide while simultaneously achieving other benefits. The current primary techniques of CO_2_ conversion are thermal catalysis, chemical catalysis, electrocatalysis, photocatalysis, enzyme catalysis, and plasma catalysis [5,6,7]. In the selection of CO_2_ conversion techniques, the selectivity is the most important consideration and the critical factor for practical applications of CO_2_ conversion, which can influence both the reaction yield and the purity of the product. Owing to its remarkable substrate specificity and chemical selectivity, enzyme catalysis technology offers a sustainable and effective option for converting CO_2_. This approach replicates the metabolic process of CO_2_ in live organisms. It offers the benefits of gentle reaction conditions, an excellent reaction efficiency, and a plentiful product yield [8]. Lyases and redox enzymes play a crucial role in the conversion of CO_2_, facilitating the production of valuable substances including formic acid, methanol, and methane. Lyases, such as carbonic anhydrase (CA), have the capacity to facilitate the cleavage of chemical bonds. Oxidoreductases, such as formate dehydrogenase (FDH), have the ability to facilitate electron transport [9,10]. Nicotinamide adenine dinucleotide phosphate reduced (NADPH), nicotinamide adenine dinucleotide phosphate (NADP^+^), nicotinamide adenine dinucleotide reduced (NADH), and nicotinamide adenine dinucleotide (NAD^+^) are crucial cofactors in redox reactions [11]. Enzyme catalysis provides a green and efficient alternative for the efficient conversion of CO_2_, owing to its exceptional substrate specificity and chemo-selectivity [12,13]. Although free enzymes, particularly oxidoreductases, have outstanding catalytic activity in converting CO_2_, they are very sensitive to the reaction conditions, prone to deactivation, and challenging to recover, which result in higher expenses. Furthermore, the reduction of CO_2_ necessitates a significant amount of NADH. Free enzymes’ industrial implementation is significantly limited by these factors [14,15].

Immobilized enzyme technology enhances both the stability and reusability of enzymes, while also addressing the limits encountered in practical applications [16,17]. Hence, it is crucial to develop an immobilized enzyme technique that can efficiently facilitate the conversion of CO_2_ in order to optimize the utilization of this technology in the industrial sector.

## 2. Engineered Metal–Organic Frameworks for CO_2_ Conversion with Enzymes

Immobilized enzyme technology involves the confinement or localization of enzymes to a specific area via physical or chemical methods, while preserving their ability to catalyze reactions. The choice of immobilized enzyme carrier is crucial for optimizing the enzyme’s catalytic performance, especially in CO_2_ conversion processes [18]. Porous materials have emerged as a popular option for immobilizing enzymes due to their distinctive physical features, which enhance enzyme stability and catalytic efficiency in CO_2_ conversion reactions [18,19].

Metal–organic frameworks (MOFs) are a novel family of porous materials, generally crystalline, formed by coordination between metal ions and organic ligands. MOFs can be carefully tailored by choosing certain metal and organic ligands, allowing for fine control over properties such as pore size and surface chemistry [20]. The adjustable pore structure, large specific surface area, and ordered crystal structure of MOFs make them ideal catalyst supports, providing a high density of active sites. In addition, a MOF can also perform CO_2_ conversion by itself. The catalytic activity of metal centers in MOFs enhances the adsorption and activation of CO_2_, thereby accelerating its conversion into valuable chemicals or energy sources [21]. Compared with traditional immobilization methods, enzyme-loaded MOFs are able to achieve higher enzyme loading and improve the catalytic efficiency, making them highly effective for CO_2_ conversion [22,23,24]. Furthermore, MOFs can prevent the enzyme from undergoing conformational changes, thus ensuring the stability and activity of the enzyme molecule within the MOFs [24].

As mentioned above, several enzymes, including CA and FDHs, have been identified as key catalysts in the conversion of CO_2_ to useful chemicals. These enzymes facilitate various CO_2_ reduction pathways, with CAs accelerating CO_2_ hydration [25]. FDHs are involved in the reduction of CO_2_ to formate in enzymatic carbon conversion systems. The efficiency of CO_2_ conversion depends not only on the intrinsic properties of these enzymes but also on the method by which they are immobilized onto supports, which can influence their stability, reusability, and overall catalytic performance [10]. Moreover, during the catalytic conversion of CO_2_, enzyme activity may be affected by changes in the microenvironment of the reaction system, leading to a reduction in catalytic efficiency. Therefore, the specific enzyme and its intended application, and the characteristics of the MOFs, should be considered in selecting an appropriate immobilization method for enzyme attachment to MOFs, or composite MOF materials modified through various treatments should be used as immobilization carriers.

Economic considerations when fabricating MOF-based materials are also a critical factor when immobilizing enzymes onto MOFs. Although energy-intensive synthesis methods, such as solvothermal techniques, can increase the upfront costs of MOF production, the potential for reuse and recycling offers a distinct advantage in reducing long-term operational expenses [26]. Moreover, the prepared MOF composites have the potential to significantly enhance the CO_2_ conversion efficiency, leading to better resource utilization [27]. This not only improves the effectiveness of CO_2_ capture and conversion processes but also contributes to lowering the operational cost per unit of CO_2_ converted [28]. Therefore, MOFs strike a promising balance between initial fabrication costs and long-term efficiency gains.

### 2.1. Enzyme Immobilization Strategies Within MOFs for Catalytic CO_2_ Conversion

**Figure 2:**
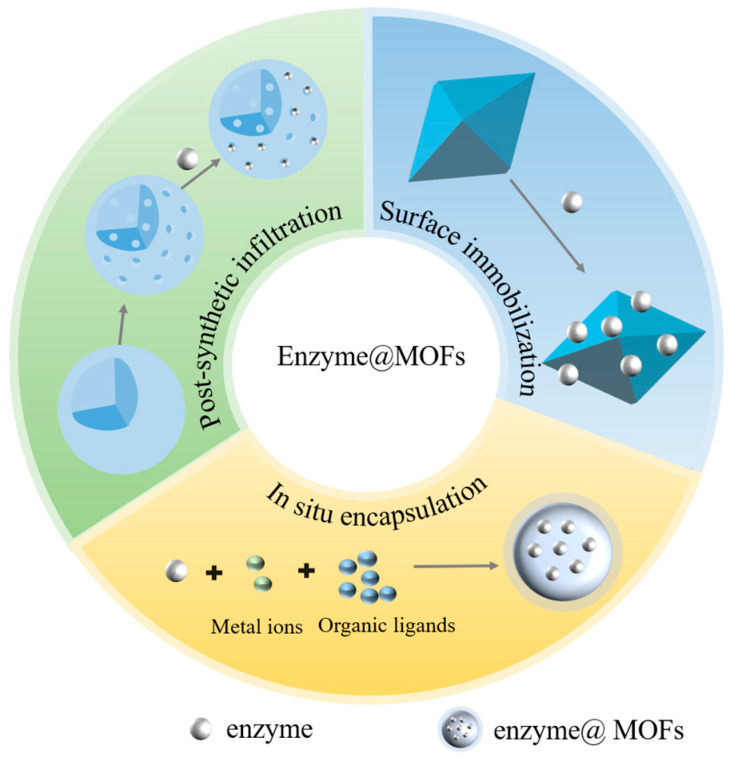
Strategies for synthesizing enzyme@MOFs.

For the purpose of catalyzing the conversion of CO_2_, researchers are currently conducting research on the use of a variety of MOFs as immobilized enzyme carriers. These materials include HKUST-1, the UIO series, the MIL series, and the ZIF series, which have the benefit of offering a safe environment that minimizes enzyme inactivation [22]. By strategically designing and optimizing the process of immobilizing, it is anticipated that the efficient conversion of carbon dioxide (CO_2_) may be achieved in industrial applications [22,29]. Typically, there are three approaches used to immobilize enzymes on MOFs: surface immobilization, post-synthetic infiltration, and in situ encapsulation (Figure 2). Surface immobilization is the process of attaching enzymes to the surface of synthesized MOFs via covalent or non-covalent interactions. Post-synthetic infiltration involves the permeation of enzyme molecules into the interior of synthesized MOFs. In situ encapsulation refers to the process of synthesizing MOFs in the presence of enzymes, resulting in the encapsulation of enzyme molecules within the crystal structure of the MOFs [30,31].

#### 2.1.1. Surface Immobilization of Enzymes on Metal–Organic Frameworks for Catalytic CO_2_ Conversion

Surface immobilization requires a substantial specific surface area and relies on the surface chemistry of MOFs. Based on the bonding process and mechanism, surface immobilization technology can be categorized into two main types: physical adsorption and covalent bonding.

Physical adsorption relies on weak interactions to attach enzyme molecules to MOF materials [32]. Covalent binding involves the tight attachment of enzyme molecules to MOFs via the formation of covalent bonds. To date, reports on the use of surface immobilization of enzymes for CO_2_ conversion remain limited [33]. This limitation is likely due to the fact that surface immobilization of enzymes on MOFs fails to take full advantage of the material’s inherent porosity, introducing challenges such as a low enzyme loading capacity, enzyme leaching, loss of catalytic activity, and limited enzyme protection, particularly in CO_2_ conversion processes [34,35]. Therefore, enhancing surface immobilization methods to improve enzyme retention and activity during CO_2_ catalysis remains a key area of research.

#### 2.1.2. Post-Synthetic Infiltration of Enzymes to Metal–Organic Frameworks for Catalytic CO_2_ Conversion

To enhance enzyme immobilization for CO_2_ conversion, synthesizing MOFs with larger pore sizes is a viable approach. However, several factors must be taken into consideration. For instance, increasing the pore size by employing longer ligands is a potential strategy for improving the CO_2_ conversion efficiency, but these materials often exhibit reduced stability and are prone to structural collapse [36]. Surfactant-induced MOF synthesis effectively creates mesoporosity through physical templating and chemical interactions, which can enhance the surface area, diffusion, and catalytic efficiency. However, precise control over synthesis conditions is necessary to maintain material strength [37].

Devised a post-synthetic infiltration technique provides a novel approach for immobilizing enzymes. The basic concept of this approach lies in the meticulous design of the pore structure of MOFs according to the three-dimensionality of enzymes, in order to accommodate a greater number of enzymes in the pores [38]. This technique allows enzyme molecules to enter MOFs under milder conditions, preserving enzyme activity and reducing leaching risks [39]. For example, Yan’s group used post-synthetic ligand substitution to modify UIO-66-NH_2_ into HP-UiO-66-NH_2_, improving the formic acid yield by 5.57 times compared to the free enzyme system [40].

It is worth noting that in addition to constructing MOFs with large pore sizes for immobilization, immobilizing enzymes within MOF materials through a one-pot synthesis or in situ method represents another approach with significant application potential in CO_2_ conversion [27].

#### 2.1.3. In Situ Immobilization of Enzymes to Metal–Organic Frameworks for Catalytic CO_2_ Conversion

Standing out from the previous approaches, the in situ immobilization technique is particularly promising for CO_2_ conversion [41]. This method involves immediately encapsulating the enzyme molecules while the MOFs are growing. It requires mild conditions to ensure biocompatibility and prevent enzyme inactivation. MOFs manufactured at low temperatures, such as L-ZIF, ZIF-8, and MIL-88, are especially well-suited to in situ encapsulating enzymes. Their narrow pores not only prevent enzyme leakage but also maintain enzyme stability, which is crucial for efficient CO_2_ conversion.

In situ encapsulation technology is primarily categorized into two methods: liquid-phase and solid-phase in situ encapsulation strategies [31].

In liquid-phase encapsulation, biomineralization and co-precipitation are the key methods of CO_2_ conversion. Biomineralization involves the encapsulation and immobilization of enzyme molecules using MOFs within a mild liquid-phase environment, mimicking natural mineralization processes. This approach employs enzymes to induce MOF formation [42]. The resulting protective shell provides structural support while preserving the enzyme’s catalytic activity and preventing degradation [43]. The co-precipitation technique, in contrast to biomineralization, entails the deposition of enzymes on the growing surface of MOFs. Here, the enzymes themselves are not involved in the nucleation process of MOFs [44]. This method has demonstrated exceptional reusability and stability in the CO_2_ conversion process.

The solid-phase in situ encapsulation strategy offers a more environmentally friendly approach to the synthesis of MOFs, by minimizing the usage of organic solvents and strong acids, while maintaining enzyme activity during CO_2_ conversion. However, there is currently a scarcity of studies on solid-phase encapsulation. Additional research and verification are necessary to elucidate the precise reaction mechanisms.

In situ encapsulation is a highly promising technique for immobilizing enzymes in MOFs, particularly for CO_2_ conversion [45]. By modifying the synthesis conditions, the enzyme’s loading capacity can be enhanced, while its stability is significantly improved. This comprehensive protection is crucial, especially under the challenging reaction conditions typical of CO_2_ conversion processes [46,47].

### 2.2. Engineered MOF Composites for Enhanced Enzyme Immobilization and CO_2_ Conversion

In contrast to free enzymes, enzyme@MOFs demonstrate an improved stability and catalytic efficacy. However, there are challenges in recycling immobilized enzymes due to the nanoscale dimensions of MOFs. Accurate and regulated integration of MOFs with functional materials becomes crucial [48,49]. This integrated strategy not only improves the activity and stability of enzymes under specific conditions but also enhances the structural stability and adaptability of MOFs [50,51]. Consequently, it promotes the application and performance of enzyme@MOFs in critical fields, especially in CO_2_ conversion. At present, there are several functional materials composited with MOFs, including chitosan/polyvinyl alcohol (CS/PVA) hydrogels, magnetite (Fe_3_O_4_) nanoparticles, and silica nanoparticles (SiO_2_ NPs) [52,53]. The purpose of the incorporation of these materials is to enhance the mechanical strength, chemical stability, and catalytic properties of MOFs, as well as to improve the efficiency of enzyme loading and the simplicity of operation.

#### 2.2.1. CS/PVA Hydrogel-MOF Composites for Enzyme Immobilization

CS/PVA hydrogels are a unique combination of chitosan and polyvinyl alcohol, which exhibit exceptional biocompatibility, degradability, and mechanical strength. The integration of hydrogel and enzyme@MOFs not only improves the stability of enzyme@MOFs but also facilitates the efficient separation of immobilized enzymes and products. Ren et al. prepared a PVA/CS/CA@ZIF-8 composite hydrogel film by encapsulating CA@ZIF-8 in PVA-CS hydrogel. The membrane greatly enhanced the loading capacity and catalytic activity of CA, while also exhibiting exceptional reusability and mechanical robustness. The activity remained above 50% after 11 cycles [53]. Wen et al. created a new type of inorganic hybrid nanoflower (CANF), which was composed of bimetallic (Cu^2+^ and Zn^2+^). The PVA/CS@CANF hydrogel film was formed by compositing CANF with the PVA-CS hydrogel network. The membrane substantially improved the conversion efficiency of CO_2_. The yield of CaCO_3_ was 9.0 times greater than that of free CA and 2.0 times greater than that of CANF. Furthermore, it exhibited exceptional stability and recyclability [54].

#### 2.2.2. Fe_3_O_4_-MOF Composites for Enzyme Immobilization

Magnetite (Fe_3_O_4_) is an oxide mineral with strong magnetism, which is predominantly composed of Fe_3_O_4_. By compositing Fe_3_O_4_ with MOFs, it is feasible to confer further magnetic characteristics to MOFs. The Fe_3_O_4_/MOF composites can be used in enzyme immobilization, which can facilitate the complete recovery of the immobilized enzyme and offers exceptional reuse capabilities [55]. Aguirre et al. have effectively constructed a recyclable biocomponent system for the catalytic conversion of CO_2_ by immobilizing FDH to Fe_3_O_4_/ZIF-8. The results indicate that the system exhibits an excellent performance in the hydrothermal stability test. The partial dissolution of ZIF-8 in the reaction system is inhibited when the concentration of the magnetic carrier reaches 10 mg/mL or higher. Furthermore, even after five cycles, the biocatalytic system exhibited a remarkable 86% retention of its activity, which effectively showcased its exceptional magnetic retrieval capability and potential for repeated use [56].

#### 2.2.3. SiO_2_-MOF Composites for Enzyme Immobilization

Silica nanoparticles (SiO_2_ NPs) exhibit exceptional advantages in numerous disciplines, including adsorption and catalyst support, owing to their high specific surface area and exceptional physicochemical stability [57]. SiO_2_-MOF composites maximize the synergistic benefits of these two materials, offering a novel approach for enzyme immobilization in the catalytic conversion of CO_2_. SiO_2_ is a support promoting the growth of MOF particles. This approach enhances material stability, prevents particle agglomeration, and facilitates particle growth [58]. Li et al. successfully synthesized a ZIF-8@FDH@SNF composite. FDH was immobilized on amino functionalization of a silica nanoflower (SNF) through electrostatic adsorption, which was subsequently decorated with a ZIF-8 layer. The immobilized catalyst effectively prevented enzyme leaching. It exhibited outstanding stability and reusability, as well as high efficiency in CO_2_ conversion [59].

In summary, the precise and controllable integration of MOFs with other functional materials not only significantly enhances the stability and catalytic efficiency of MOF@enzyme composites but also addresses challenges such as recycling difficulties, susceptibility to damage, and limited stability under specific conditions. These innovative composites show considerable potential for CO_2_ conversion, providing strong support for the advancement of an efficient and sustainable carbon cycle.

## 3. Innovative Strategies for Boosting CO_2_ Conversion Efficiency in Enzyme@MOFs

The immobilization of enzymes on MOFs is regarded as a successful approach to improve the activity and stability of enzymes, which increases the efficacy of CO_2_ conversion. However, the efficiency of CO_2_ conversion is influenced not solely by the catalytic performance of the immobilized enzyme but also by the solubility of CO_2_ in the catalytic system, the mass transfer efficiency of CO_2_ at the gas–solid interface, and the concentration of coenzyme. To address these issues, researchers have conducted extensive investigation focused on improving the efficiency of CO_2_ utilization, coenzyme regeneration, and substrate mass transfer. The findings are comprehensively reviewed in this paper.

### 3.1. Boosting CO_2_ Utilization to Improve the Conversion Efficiency in Enzyme@MOFs

#### 3.1.1. Strategies for Modifying MOFs to Enhance CO_2_ Adsorption for an Improved Conversion Efficiency in Enzyme@MOFs

MOFs are ideal materials for gas storage, due to their notably large specific surface area. The adsorption capacity of CO_2_ can be enhanced by delicately tuning the dimensions and configuration of MOF pores through the meticulous design of organic ligands and metal nodes [60,61]. Currently, a number of MOFs, such as ZIF-8, HKUST-1, MIL-101, and UIO-66-NH_2_, have exhibited a unique capacity to adsorb CO_2_ [62,63,64]. The UIO series, in particular, exhibits a well-developed micropore structure and a substantial specific surface area. Furthermore, the kinetic diameter of CO_2_ molecules is in close alignment with the pore dimensions, which enables the specific adsorption of CO_2_ [65].

The adsorption capacity of CO_2_ by MOFs can be greatly improved through the introduction of functional groups. Specifically, MOFs, which possess open metal sites, have the capability to establish a strong interaction with CO_2_ molecules, enhancing the efficacy of adsorption. Fernando et al. successfully synthesized Ce-UiO-66-NH_2_ with 84% amino functionalization, which exhibited excellent physicochemical properties. Compared to Ce-UiO-66, Ce-UiO-66-NH_2_ demonstrated a 63% higher CO_2_ absorption rate and an 84% higher CO_2_/N_2_ selectivity. These findings suggest that MOFs functionalized with groups on either the linker or metal sites exhibit an enhanced CO_2_ absorption capacity and selectivity. Moreover, Ce-UiO-66-NH_2_ showed a relatively low water vapor absorption rate while maintaining excellent structural stability after gas and steam adsorption, highlighting its strong potential as a CO_2_ adsorbent [66]. This functionalization strategy can significantly improve the CO_2_ capture capacity of MOFs, due to the strong interaction between nitrogen atoms with high electronegativity and the positively charged carbon atoms in CO_2_ molecules [67,68]. Polyethyleneimine (PEI) is a cationic polyelectrolyte, which comprises primary, secondary, and tertiary amino groups. It has the ability to interact with a variety of functional groups on enzymes or MOFs [69,70]. Lin’s group attempted to modify MIL-101 with varying concentrations of PEI. The results revealed that the CO_2_ adsorption capacity of the modified MIL-101 increased considerably under atmospheric pressure, despite a decrease in its surface area and porosity. The adsorption capacity reached 4.2 mmol·g^−1^ at 0.15 bar and 25 °C when the PEI concentration approached 100 wt% [71]. Li et al. further functionalized MIL-101 with hexamethylenediamine (HMD), cystamine, and PEI. PEI(100)-MIL-101 demonstrated the highest CO_2_ adsorption capacity of 8.25 mmol/g, which was 4.4 times that of the original MOFs. The structure of MIL-101 was minimally affected by amine functionalization, suggesting that this method was highly compatible with MOF structures [72].

**Figure 3:**
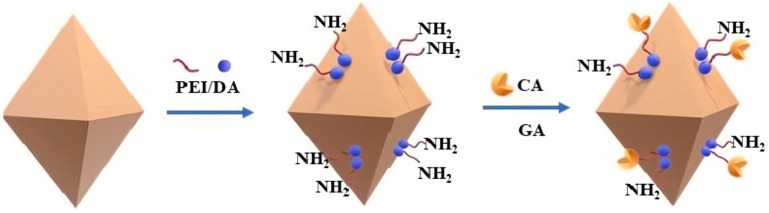
Schematic illustration of the preparation process for PEI/PDA-MOF-808 and CA@PEI/PDA-MOF-808 [19].

Due to the small pore size and sparse functional groups on the surface of MOF-808, the loading capacity of enzymes immobilized on MOF-808 is low. This problem can be resolved by functionalizing MOF-808 with an amino group. Furthermore, these amino groups in MOF-808 have the potential to react with CO_2_ to produce carbamate and bicarbonate during the CO_2_ capture and conversion process. Consequently, the adsorption capacity of CO_2_ is substantially raised. Xv et al. modified MOF-808 by co-depositing PEI and polydopamine (PDA) (Figure 3). The CO_2_ capture capacity of CA was improved by immobilizing it with the prepared PEI/PDA-MOF-808. The total yield of CaCO_3_ reached 294.0 mg in eight consecutive cycles of CO_2_ conversion, which was 92 times that of free CA. Additionally, the CA immobilized to PEI/PDA-MOF-808 demonstrated exceptional thermal stability, pH stability, storage stability, and reusability, even in the presence of severe acidic conditions [19].

HKUST-1 is an additional variety of MOF material that exhibits an exceptional capacity for CO_2_ adsorption. Nevertheless, the hydrophilicity of HKUST-1 can result in its irreversible structural alterations in the presence of water. This attribute restricts its potential for use in the field of enzyme immobilization [73,74]. Stearic acid (SA) is a type of fatty acid that is composed of long-chain alkyl and carboxylic groups. In addition to its exceptional biocompatibility and stability, it also exhibits hydrophobic properties. By functionalizing MOFs with SA, the pore structure and surface chemical properties of MOFs can be improved, which results in the enhancement of its thermal and chemical stability [75,76]. Yan and Li initially modified HKUST-1 with SA using an in situ hydrothermal procedure. The crystal structure and morphology of the modified HKUST-1 remained nearly unaltered, while its specific surface area experienced a substantial increase. Additionally, SA functionalization enhanced the hydrophobicity and stability of HKUST-1 in aqueous solutions. The hydrophobic surface of modified SA@HKUST-1 had the potential to enhance CO_2_ absorption capacity, providing a novel approach to CO_2_ catalytic conversion [76]. Rouf et al. employed SA for post-synthetic modification of HKUST-1 to enhance its stability in aqueous solutions. This modification enabled HKUST-1 to function as an effective support for FDH in the efficient conversion of CO_2_ to formic acid [77].

#### 3.1.2. Strategies for Enhancing CO_2_ Solubility to Improve the CO_2_ Conversion Efficiency in Enzyme@MOFs

The low CO_2_ conversion rate is also influenced by the limited solubility of CO_2_ in aqueous solution. Ionic liquids (ILs) are solutions that are composed of ions and remain in a liquid state at a normal ambient temperature. They are capable of efficiently capturing CO_2_ through electrostatic interaction, van der Waals interaction, hydrogen bonding interaction, and other interactions [78,79]. Ionic liquids that contain amino groups are currently being developed for the purpose of CO_2_ capture. CO_2_’s solubility in ILs may exceed 0.5 mmol/mol ILs [80]. The first instance of CO_2_ conversion to methanol in ILs was reported by Zhang et al. Choline and amino-acid-based ILs (i.e., [CH][Glu], [CH][Pro], [CH][Gly], and [CH][His]) were developed and manufactured. The yield of methanol in a 20% [CH][Glu] IL was approximately 3.5 times greater than that in a pure water system [81]. Despite the substantial potential of ILs in CO_2_ capture, their use in large-scale applications is limited by the expensive costs of their preparation.

In addition to the direct increase in the solubility of CO_2_ in water, it can also improve its content in water through the hydration reaction of CO_2_. CA has the ability to catalyze the formation of bicarbonate by reacting with water and CO_2_ [24]. Consequently, the efficient conversion of CO_2_ and the solubility of CO_2_ in water can be significantly enhanced through the integration of CA in enzyme@MOFs. The adsorption of CO_2_ in the catalytic system was considerably increased by the introduction of CA during the electrocatalytic reduction of CO_2_ to formic acid, as demonstrated by Liu et al. This thereby effectively increased the yield of formic acid [82].

#### 3.1.3. Strategies for Maximizing CO_2_–Enzyme Contact to Improve the CO_2_ Conversion Efficiency in Enzyme@MOFs

The prevalent approach in current research on enzyme-catalyzed CO_2_ reduction is to disperse the immobilized enzyme in an aqueous solution and introduce CO_2_ bubbles. Nevertheless, a more effective approach is to position the enzyme at the gas–liquid interface in order to obtain optimal contact between CO_2_ and the enzyme. In the field of CO_2_ capture technology, gas–liquid film contactors are a promising method. The film functions as an interphase transfer interface, enabling the effective transmission of mass and contact between the liquid and gas phases. An adsorption method was employed by Liu et al. to attach CA to the MIL-160/Al_2_O_3_ film. This immobilization enabled CO_2_ molecules to directly interact with the enzyme’s active site, thereby circumventing the restriction of CO_2_’s inadequate solubility in water [83]. In addition, the hydrophilic microenvironment provided by MIL-160 was conducive to maintaining the enzyme’s biological activity. The Rh complex was precisely anchored to the ligand of MIL-125-NH_2_ by Lin et al. NADH regeneration was accomplished through the utilization of the photoenzymatic approach, which coupled with the immobilized enzyme system to generate formic acid. The conversion of NAD⁺ to NADH was expedited by the transfer of electrons between MOFs and Rh complexes under light conditions. Furthermore, the efficient three-phase contact system of CO_2_ (gas), H_2_O (liquid), and immobilized enzyme (solid) was successfully established to facilitate the enzyme-catalyzed reaction by immobilizing FDH to the hydrophobic layer of the membrane. The membrane served as a support for enzyme-catalyzed reactions, in addition to acting as a physical barrier to form a unique gas–liquid interface. Therefore, the immobilized enzyme’s recovery rate was substantially enhanced [84].

### 3.2. Efficient Coenzyme Regeneration to Enhance CO_2_ Conversion in Enzyme@MOFs

The involvement of hydrogen and electrons is crucial in the process of CO_2_ reduction. Particularly in bioenzyme-catalyzed processes, natural coenzymes are frequently required to function as electron acceptors and hydrogen donors. For example, FDH is an enzyme that catalyzes the conversion of formate (HCOO^−^) to CO_2_. The reaction process is HCOO^−^ ⇌ CO_2_ + H^+^ + 2e^−^. Natural coenzymes such as NAD^+^ and NADP^+^ are involved in this reaction as electron acceptors. Under mild conditions, FDH can also utilize reduced coenzymes, such as NADH or NADPH, as electron donors to facilitate their reverse process. This reaction could reduce CO_2_ to formate (CO_2_ + H^+^ + 2e^−^ ⇌ HCOO^−^) [85,86,87].

Injecting electrons directly into an enzyme’s active center is a highly efficient method of providing reducing power. Unfortunately, many enzymes do not have internal metal cluster chains to aid in electron transfer, which restricts the widespread use of this method. In the CO_2_ reduction process catalyzed by FDH, NADH serves as an important sacrificial agent, providing the necessary hydrogen and electrons for the reduction reaction [86]. However, the high cost of NADH consumption limits its feasibility for industrial-scale application. Furthermore, the NAD^+^ generated in the process may inhibit further formate production. Therefore, regenerating NAD+ into NADH not only enables the sustainable recycling of NADH but also reduces product inhibition, which increases the yield [88].

Recently, scientists have thoroughly investigated many approaches to regenerate NADH, such as chemical regeneration, photochemical regeneration, electrochemical regeneration, and enzymatic regeneration. Table 1 presents the application of different strategies for coenzyme regeneration in the process of CO_2_ reduction using enzyme@MOFs, and it compares the yield performance levels of production under different regeneration conditions.

#### 3.2.1. Chemical Regeneration of Coenzyme to Improve the CO_2_ Conversion Efficiency in Enzyme@MOFs

**Figure 4:**
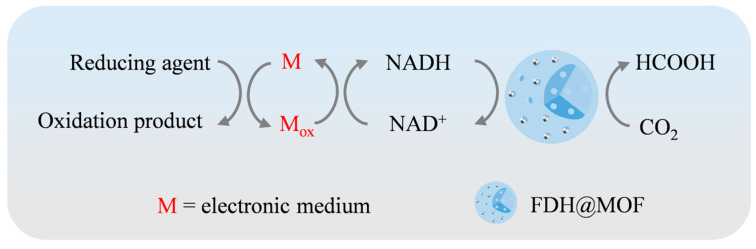
Schematic diagram of the chemical regeneration reaction mechanism.

The purpose of coenzyme chemical regeneration is to facilitate a redox reaction catalyzed by a specific chemical catalyst in the CO_2_ reduction system. In this reaction, the reducing agent supplies a sufficient number of electrons and protons (Figure 4). The most widely used inorganic salt reducing agents include sodium formate (HCO_2_Na), sodium dithionite (Na_2_S_2_O_4_), and sodium borohydride (NaBH_4_) [101,102]. In addition, the catalyst can transfer the electrons produced by the oxidizing reducing agent to the coenzyme, greatly enhancing the rate of NADH regeneration. In the quest for efficient catalysts, organometallic catalysts containing elements such as ruthenium, rhodium, and iridium show great potential to regenerate NAD^+^. Fard et al. prepared Rh complexes composed of [(Cp*)RhCl]_2_^2+^ (pentamethylcyclopentadienyl rhodium (III) chloride dimers) with various ligands (2,2′-bipyridine, 1,10-phenanthroline, and their derivatives) and compared their reactivity in chemical coenzyme regeneration. Among them, [Rh(Cp*)(bpy)Cl_2_] (bpy = 2,2′-bipyridine) showed the highest catalytic efficiency, with the NADH yield reaching nearly 100% when sodium formate was used as the reducing agent [89].

Chemical regeneration technology could quickly achieve, with the help of appropriate catalysts, the conversion of NAD^+^ to NADH, which has the advantages of high efficiency, flexibility, and wide applicability [89]. However, chemical regeneration necessitates the employment of noble metal catalysts and large amounts of water-soluble reducing agents, which might result in enzyme inactivation as well as difficult recycling reducing agents [103]. This limits the economic viability of the method at an industrial level. Therefore, more work needs to be carried out to develop a greener strategy of coenzyme regeneration that can be applied more effectively to NADH regeneration, especially in the FDH-mediated conversion of CO_2_ to formic acid.

#### 3.2.2. Electrochemical Regeneration of the Coenzyme to Improve the CO_2_ Conversion Efficiency in Enzyme@MOFs

**Figure 5:**
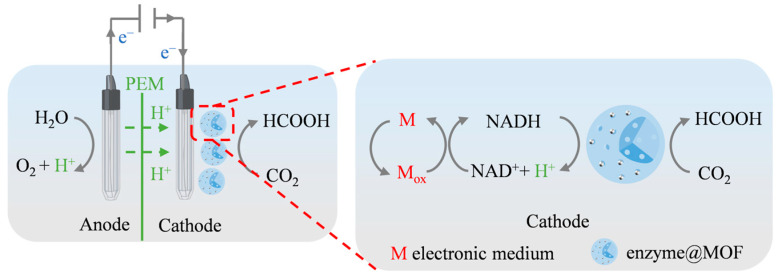
Schematic diagram of the electrochemical regeneration reaction mechanism.

In cells, the electron transfer chain (ETC) is a key link in the production of adenosine triphosphate (ATP) during cell respiration. NADH and flavin adenine dinucleotide (FADH_2_) are the coenzymes that play a vital role in the ETC. These coenzymes accelerate oxidative phosphorylation by transferring electrons to downstream carriers in the chain, promoting ATP synthesis [104]. Enzyme electrosynthesis (EES) refers to a bioelectrocatalytic technique. Enzymes are propelled to catalyze using electrons captured by the cathode. These electrons can enter the catalytic center of oxidoreductases directly from the electrode or indirectly through an electron mediator or coenzyme (Figure 5) [105,106]. Therefore, the choice of electrode potential, electrode material, and electron mediator can significantly affect product purity. CO_2_ reduction and NADH regeneration can be accomplished with an effective method called the CO_2_ enzyme electrocatalytic reduction system [107]. In this system, electron mediators including Methyl Viologen (MV^2+^), Neutral Red (NR), and CpRh(bpy)Cl_2_ and its derivatives are often used.

NR, as an efficient electron mediator, exhibits excellent redox activity. Its midpoint potential (Em) can significantly positively shift under specific conditions, thereby enhancing its electron transfer efficiency [108]. Li et al. successfully integrated the synthesized ZIF-8@FDH@SNF immobilized enzyme with an electrocatalytic coenzyme regeneration system. Efficient enzymatic electric reduction of CO_2_ was achieved by employing NR as an electron mediator. The electrochemical enzymatic system significantly improved the formic acid production efficiency. The yield of formic acid reached 1.38 mM within 3 h, which was 3.1 times greater than that of the free enzyme system without the NADH regeneration mechanism, indicating considerable performance improvements. In addition, NR was often used to modify the electrode surface, to enhance the efficiency of electron transfer between the electrode and the enzyme [59]. Liu et al. developed an effective CO_2_ enzyme electrocatalytic device. The system introduced PEI modification technology, which increased both the stability of immobilized FDH on SBA-15 and the adsorption capacity of CO_2_. The effective electrochemical regeneration of NADH from NAD⁺ was accomplished utilizing an NR-modified copper electrode. After 3 h of continuous reaction, the yield of formic acid was 1.118 mM, which was 3.7 times higher than that of the free enzyme system [33].

CpRh(bpy)Cl_2_ is an organometallic complex composed of rhodium (Rh) as the central metal ion, a pentamethylcyclopentadienyl (Cp) ligand, a chloride ion (Cl^−^), and 2,2′-bipyridine (bpy). This complex has a unique electron configuration and chemical properties, making it an ideal electron transport medium for various chemical reactions [106]. Chen et al. constructed a bioelectrocatalytic system based on NU-1006 co-immobilizing FDH and a modified Rh complex. The conversion rate of NAD⁺ reached 90% after 1 h of applying the potential, and formic acid production was doubled when compared with that of free enzymes [92]. Zhang et al. prepared a carboxylated Rh complex (CpRh(bpydc)Cl_2_) using 2,2′-bipyridine-5,5′-dicarboxylic acid (bpydc) as the coordinating ligand and immobilized them on the cathode surface. The heterophase catalytic regeneration of NADH was accomplished through electrochemical methods. Additionally, by coupling the multi-enzyme cascade system in ZIF-8 with the electrocatalytic regeneration of NADH, the CO_2_ conversion rate was significantly improved [81].

MV^2+^ can also be used as an electron acceptor and transfer catalyst in the electrocatalytic redox reaction [109]. Liu’s team developed an nFDH@ZIF-90@CA double-enzyme microreactor, where FDH was first encapsulated in polyacrylamide microcapsules and then covalently bonded to ZIF-90. This dual protection strategy considerably enhanced the chemical, thermal, and storage stability of the immobilized enzyme. In the enzyme electrocatalytic system using MV^2+^ as the electron mediator, the CO_2_ reduction capacity was markedly improved, with the rate of NADH regeneration increased. After 3 h of reaction, the formic acid yield reached 2.917 mM, which was 8.9 times greater than that of the free enzyme system. This offered an efficient and sustainable solution for converting CO_2_ into formic acid [82]. Because of its unique chemical structure and properties, MV^2+^ is widely used as an electron mediator in electrocatalysis. However, due to the biological toxicity of MV^2+^, it is necessary to pay great attention to safety and stability factors during its use.

Electrocatalysis technology shows great potential in NADH regeneration, which is considered a promising strategy. Compared to alternative means of NADH regeneration, electrochemical techniques stand out for their higher efficiency, lower cost, and environmental friendliness. Nevertheless, there are also additional challenges with the electrocatalytic process, such as the weak specificity and high cathode potentials required.

#### 3.2.3. Photochemical Regeneration of the Coenzyme to Improve the CO_2_ Conversion Efficiency in Enzyme@MOFs

Photocatalysis is a process that uses light energy to excite photocatalysts, generating electron–hole pairs that drive chemical reactions. Upon the absorption of photons by the photocatalyst, electrons are excited from the valence band to the conduction band, where they participate in reduction reactions. The holes remain in the valence band to drive oxidation reactions [110]. Photocatalytic processes have been widely applied in energy conversion, environmental protection, and synthetic chemistry.

Photochemical regeneration of NADH is a smart technique that emulates the natural photosynthesis process of plants by harnessing light energy to facilitate the conversion of NAD^+^ to NADH. In CO_2_ conversion, the photochemical regeneration of NADH relies on three essential components, that is, the photosensitizer, the electron mediator, and the electron donor [111,112]. The primary role of the photosensitizers is to collect light energy and convert it into excited states with increased energy and an enhanced redox capacity, facilitating electron transfer [113,114]. Some photosensitizers, such as porphyrins and their derivatives, can more effectively mimic the photosynthesis process in nature due to their molecular structure, which is similar to chlorophyll. Porphyrin rings, acting as the center of light energy collection, efficiently absorb photons and convert them into electrons that can be transferred to the electron mediator [115]. Because of the energy mismatch between the excited photosensitizer and NAD^+^, an electron mediator is usually required to facilitate the regeneration of coenzymes. Currently, the rhodium-based complex (CpRh(bpy)Cl_2_) and its derivatives are attracting attention as efficient electron mediators. They are capable of capturing photogenerated electrons produced by the photocatalyst in photocatalytic or enzyme-catalyzed reactions and transferring these electrons to the target molecule (such as NAD^+^) via electron transfer pathways. Thus, NADH regeneration or other reduction reactions can be achieved [116]. As for electron donors, common substances include water or triethanolamine (TEOA).

**Figure 6:**
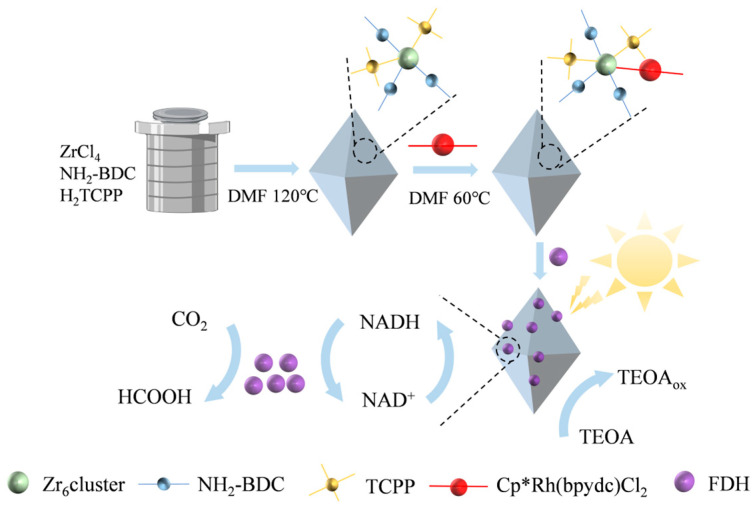
Schematic illustration of the artificial photosynthesis strategy.

During the synthesis of UiO-66, the introduction of the photosensitive ligand Tetra(4-carboxyphenyl)porphine (TCPP) not only enhances the catalytic active sites of the enzyme but also improves the overall photoelectric performance [117,118]. For the first time, Xing et al. constructed an efficient semi-artificial photosynthetic system. They applied a biligand UiO-66 (2,2′-Bipyridine-3,3′-dicarboxylic Acid (BPDC) and TCPP) containing a pre-loaded Rh complex to achieve in situ regeneration of NADH and immobilization of FDH. As an electron bridge, the Rh complex effectively improved electron transfer efficiency. Compared to UiO-66-NH_2_, Rh-H_2_TCPP-UiO-66-NH_2_ showed significantly enhanced photocatalytic NAD⁺ reduction activity, and the conversion rate of CO_2_ to formic acid increased from 150 μg/mL to 254 μg/mL (Figure 6). This result demonstrated the method’s significant potential for efficient CO_2_ conversion [94].

ZIF-8 is widely used as a carrier for immobilized enzymes due to its unique pore structure, highly controllable framework, and easy functionalization. Zhou et al. introduced TCPP into ZIF-8 to construct a photocatalytic multi-enzyme cascade biomimetic carbon sequestration system. Under optimal conditions, the NADH reduction yield reached 75.04%, which was higher than that of free TCPP. This method implemented the reuse of the photocatalyst TCPP and significantly increased the output of formaldehyde, which provided a novel approach to improve the efficiency of NADH regeneration through material immobilization or modification of photosensitizers [95].

NU-1006 features a distinctive stratified pore structure and demonstrates a high affinity for enzymes. As an effective photosensitizer, it can produce singlet oxygen under LED illumination. Chen’s group developed a semi-artificial photosynthesis system. Immobilized FDH was used as a catalyst, and the electronic medium CpRh(bpy)Cl_2_ was connected to the metal node of NU-1006. Under irradiation, NADH was regenerated at a rate of about 28 mM·h^−1^, and the formic acid yield was 79.34 mM·h^−1^ [119]. Gu et al. integrated 1-(carboxynonyl)-1′-methyl-[4,4′-bipyridine]-1,1′-diium (CNMV) and FDH into NU-1006. The photogenerated electrons migrated to the metal nodes through the conjugated skeleton of the ligands, which resulted in ultrafast photogenerated electron transfer between CNMV and FDH. The reaction kinetics were 190 times those of the free NADH system [96].

The inorganic photocatalyst–enzyme system is a crucial framework for the photoreduction of carbon dioxide into valuable chemicals and fuels. The regeneration of NADH is essential for sustaining continuous photoenzyme-catalyzed CO_2_ reduction. Nevertheless, the limited efficiency of electron transport kinetics during photoexcitation hampers the regeneration of light-driven NADH, which significantly constrains the total catalytic efficiency [98,120]. When the distance between the electronic medium and FDH is too short, the electronic medium can be activated by the functional groups in FDH. However, the structure of FDH can also be damaged by the by-products of photocatalytic reaction, leading to stability and catalytic activity decreases. Setting the proper distance between the electronic medium and FDH cannot only ensure the quick transport of the reaction intermediates but also avoid the deactivation of the metal center and photosensitive damage to FDH during the CO_2_ conversion process. Thus, the catalytic efficiency of FDH could be improved [121]. Pegah et al. immobilized Rh complexes and FDH into Janus-type DNA nanosheets (NSs) with double-sided heterogenic DNA sequences, respectively. Based on the surface selectivity of DNA, the NSs conjugated the Rh complex and FDH to form four different configurations. The turnover number (TON) of formic acid produced by a cascade reaction system using free FDH and NS1 was 1360, which is the highest value reported to date for Rh-based photocatalyst/enzyme coupled systems. The reactivity of NS3 was markedly superior to that of NS4, suggesting that spatial organization had a substantial impact on the total catalytic activity. Hence, the compartmentalization of the photocatalyst and the enzyme was a potent approach to enhance the efficiency of CO_2_ conversion. Photochemical coenzyme regeneration technology provides several benefits, including cost-effectiveness, eco-friendliness, and the use of renewable resources. Nevertheless, this technique encounters certain obstacles, such as the durability of the photosensitizer, complications in recycling, insufficient stability of the photocurrent, and the need to effectively separate by-products. Additionally, prolonged exposure to light can greatly diminish the activity of enzymes. In order to address these difficulties, a compartmentalized reactor can be utilized to carry out enzyme-catalyzed reactions in a controlled environment. For instance, the enzymatic process occurs in the presence of light, and the regeneration of NADH also takes place under light [121].

#### 3.2.4. Enzymatic Regeneration of the Coenzyme to Improve the CO_2_ Conversion Efficiency in Enzyme@MOFs

Enzymatic regeneration technology can achieve the efficient regeneration of NADH by using a secondary enzyme system, which has been widely used in industrial production. The enzymes commonly used for NADH regeneration include glutamate dehydrogenase (GDH(Glu)), formate dehydrogenase (FDH), and glucose dehydrogenase (GDH(Glc)). Among them, GDH(Glu) has excellent stability and cost-effectiveness, giving it the potential for application in NADH regeneration systems [122].

Ren et al. developed a multi-enzyme Co-IMR system. NADH was effectively immobilized in ZIF-8 via ion exchange between polyethylenimine (PEI) and NADH. The regeneration of NADH was accomplished by encapsulating GDH (Glu) in ZIF-8. Compared to free multi-enzyme systems, the Co-IMR system achieved a significant increase in formate production, of 4.6 times [90]. Zhu et al. also employed GDH (Glu) for NADH regeneration in a multi-enzyme immobilization cascade system. The system showed very high catalytic activity, and the methanol yield increased from 0.975 μmol·h^−1^ to 2.383 μmol·h^−1^ [91].

Enzymatic NADH regeneration is strongly recommended due to its exceptional selectivity, efficiency, and environmental compatibility. Nevertheless, this technique continues to encounter obstacles such as limited efficiency in transferring mass to the substrate, suppression of product formation, and inadequate stability of enzymes. Therefore, the co-immobilization of coenzyme-regenerating enzyme and CO_2_ reductase to construct a multi-enzyme cascade system has become the focus of research.

### 3.3. Strategies for Multi-Enzyme Co-Immobilization to Enhance the CO_2_ Conversion Efficiency in Enzyme@MOFs

In nature, many biochemical reactions are catalyzed by multi-enzyme cascade systems (MECSs), which consist of a series of highly organized enzymes. Compared with typical stepwise synthesis, cascade reactions eliminate the tedious process of separating and purifying reaction intermediates, hence attaining greater yields [123]. Furthermore, the cascade reaction can easily control the orientation of the reaction via the synergistic effect of enzymes in the system, in order to achieve the desired outcome [124]. The conversion of CO_2_ to CH_3_OH is a typical cascade reaction. By using formate dehydrogenase (FDH), formaldehyde dehydrogenase (FaldDH), and alcohol dehydrogenase (ADH), CO_2_ is gradually reduced to formic acid, formaldehyde, and methanol. NADH/NAD^+^ serves as a vital cofactor in this cascade, playing a critical role in ensuring the efficient functioning of the process. Thus, the cascade reaction system frequently incorporates coenzyme regeneration enzymes [88,90]. Furthermore, in many studies, carbonic anhydrase (CA) is also integrated into this enzymatic cascade system, facilitating the rapid hydration of CO_2_ to bicarbonate. Implementing this method can enhance the system’s capacity to trap CO_2_, hence optimizing the efficiency of the entire cascade process [82].

The practical application of the free multi-enzyme cascade system is hindered by challenges related to the recovery process and the high associated costs. Additionally, maintaining the activity and stability of diverse enzymes is also challenging due to the distinct features of each enzyme. The aforementioned issues can be resolved by implementing a suitable enzyme immobilization technique [125]. Co-immobilizing multi-enzymes cannot only extent lifespan of the enzymes but also create a stable microenvironment, greatly enhancing the stability and catalytic effectiveness of the enzymes. Moreover, the strategic arrangement of many enzymes inside a single system, in close proximity to one another, can significantly enhance the catalytic synergy [124]. Given the sequential nature of the cascade reaction, the multi-enzyme co-immobilization technique necessitates the meticulous management of enzyme localization and orientation in order to maximize the efficiency of the substrate route [126,127,128,129]. Currently, the techniques employed for co-immobilizing the enzyme cascade reaction of CO_2_ reduction mostly consist of random co-immobilization, compartment co-immobilization, and position co-immobilization. These approaches are designed to enhance the contact and reaction pathway between enzymes and substrates in order to obtain the optimal catalytic efficiency. Table 2 provides a comprehensive summary of the use of multi-enzyme co-immobilization techniques in the catalytic conversion of CO_2_ into high-value goods, as seen in recent research.

#### 3.3.1. Random Co-Immobilization Strategy for Enhanced CO_2_ Conversion in Multi-Enzyme@MOFs

The most basic technique for co-immobilizing a multi-enzyme is random co-immobilization. This method allows multiple enzymes to be immobilized on the surface or within the pores of MOFs through physical adsorption, encapsulation, covalent bonding, or chemical crosslinking [133]. The construction of this co-immobilized multi-enzyme system involves mixing several enzyme solutions to facilitate cross-linking between the enzymes, and sequentially attaching them to certain supporting materials. This approach aims to mimic the structure of natural free multi-enzyme complexes and enhance their reusability [134].

Ren et al. employed a co-precipitation method to encapsulate CA, FateDH, GDH (Glu), and NADH in ZIF-8, in order to create a nanoscale multi-enzyme reactor. The employment of CA substantially promoted the solubility of CO_2_ in the reaction system, hence enhancing the reactor’s capacity to convert CO_2_ into formate. Compared to the free multi-enzyme system, the formate yield of the system increased by 4.6 times, while maintaining a productivity level of over 50% after eight cycles [90]. To address the problems of low water solubility of CO_2_ and the high cost of NADH, Zhang et al. devised a very effective bioelectrocatalytic system. In this system, FDH, FaldDH, and ADH enzymes were in situ encapsulated within a ZIF-8 framework, and a Rh complex-modified electrode was introduced to achieve efficient NADH regeneration. The results demonstrated that the electrode was capable of converting NAD^+^ to NADH with a high degree of efficiency. At a voltage of -0.7 V, the production of methanol reached a maximum of 0.742 mM, which was 12 times greater than that of the enzyme system without any modifications [93].

Recently, there has been growing interest in utilizing composite membrane materials derived from MOFs as a viable alternative to particle carriers. Chai et al. used a combination of polydopamine/polyethylenimine (PDA/PEI) layers to modify the surface of polypropylene film (PP film) in order to improve the in situ formation of polyenzym@MOFs. PDA had the ability to adhere to hydrophobic polymers, which had a low surface energy, such as polypropylene. PEI was a polymer consisting of a significant quantity of amino acids, which could enhance the rate of deposition and prevent the aggregation of PDA. As a result, a thin and uniform bioadhesive layer of PDA/PEI was formed. The catechol groups in PDA could attach Zn^2+^ by ordination, aiding in the synthesis of CA&FDH@ZIF-8 on the membrane’s surface. When the biocatalytic membrane was placed in a gas–liquid membrane contactor for 4 h, the formic acid yield reached 22% (5.6 μmol) [130].

The utilization of random co-immobilization can effectively reduce enzyme inactivation by virtue of its straightforward implementation. It has become the primary strategy to improve the overall catalytic activity and stability of enzymes. However, in the multi-enzyme cascade system prepared by this method, the in situ consumption of intermediate products may lead to the inhibition of enzyme activity, which may further decrease the CO_2_ conversion efficiency. In addition, CO_2_ as a substrate must permeate the supporting material from the solid–liquid interface to participate in further reactions. The mass transfer routes of the resultant intermediates are not consistent. This indicates that the substrate route has not been fully optimized, which eventually has a substantial impact on the yield of CO_2_ conversion. Furthermore, the method continues to face difficulties in regulating the proportion of immobilized enzymes and the distribution of surface active groups [129].

#### 3.3.2. Compartmentalized Co-Immobilization Strategy for Enhanced CO_2_ Conversion in Multi-Enzyme@MOFs

Compartmental co-immobilization technology mimics the precise organization and synergistic mechanisms of enzymes within their native cellular surroundings. Although it is challenging to completely recreate the intricate cellular environment, compartmental co-immobilization offers several advantages. It improves the stability of the enzyme system by constructing a strong and structure-sensitive multi-compartment system. Additionally, it creates an optimal platform for efficient and specific multiple-enzymatic cascades [126,128].

**Figure 7:**
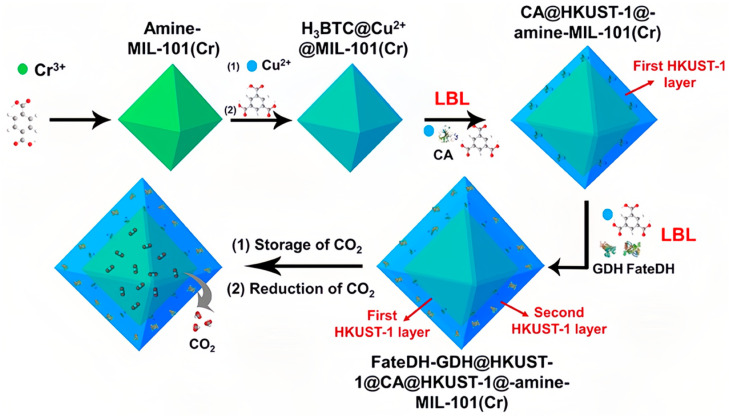
Schematic illustration of the preparation of HKUST-1@amine-MIL-101(Cr)-based multi-enzymes for the reduction of adsorbed CO_2_ [72].

Up to now, only a limited number of research studies have investigated the application of compartment co- immobilization technology to facilitate the simultaneous fixation of CA and FDH for the conversion of CO_2_. Li et al. developed an innovative MOF framework. The scaffold consisted of amine-functionalized MIL-101(Cr) as the core and was coated with two layers of HKUST-1. It not only acted as an adsorbent of CO_2_ but also achieved the compartment co-immobilization of multiple enzymes through a layer-by-layer self-assembly technique (Figure 7). Amine-functionalized MIL-101(Cr) exhibited high efficiency in adsorbing CO_2_. The CA located inside the inner layer of HKUST-1 converted the emitted CO_2_ into HCO^3−^. Subsequently, the HCO^3−^ ion underwent straight migration to the outer layer of HKUST-1, which encapsulated FDH, and was subsequently transformed into formic acid. Additionally, the GDH on the outer MOF facilitated NADH regeneration. This novel approach harnessed adsorbed carbon dioxide (CO_2_) as the substrate and optimized the substrate transportation pathway, resulting in an efficient catalytic performance and continuous regeneration of NADH. Additionally, the operational stability and reusability of the immobilized enzyme were significantly improved [72].

Specifically, the implementation of compartmentalization technology relies on various meticulously engineered v carrier materials. The materials must be skillfully assembled into many distinct functional compartments, which can be used to immobilize different types of enzymes independently. This spatial compartment immobilization technique can successfully prevent mutual interference between distinct enzymes and reduce the negative influence of inhibitors or enzyme degradants on enzyme activity. This ensures the stability and permanence of enzyme activity. Hence, due to its distinctive benefits, the compartment co-immobilization technique has demonstrated significant potential in enhancing the catalytic efficacy and durability of multi-enzyme systems. Nevertheless, the immobilization process operation is intricate and comes with a greater cost, which continues to limit its practical use.

#### 3.3.3. Sequential Co-Immobilization Strategy for Enhanced CO_2_ Conversion in Multi-Enzyme@MOFs

Compartmental co-immobilization involves separating enzymes into distinct regions to minimize interference and optimize individual reaction conditions. This method is particularly suitable for applications requiring enzymes to function in different environments [126]. Sequential co-immobilization is a way of immobilizing multi-enzymes in a systematic manner. Distinguishable from the compartmental co-immobilization approach, sequential co-immobilization emphasizes the progressive immobilization and synergistic work of enzymes [72]. It replicates the arrangement of multi-enzyme cascade systems in biological organisms, which are organized in a precise sequence and exhibit an extremely high catalytic efficiency. Milene et al. employed mercaptopropyl functionalized silicic (MCF) as the carrier to consecutively immobilize FDH, FaldDH, and ADH for the purpose of catalyzing the conversion of CO_2_. The co-immobilized enzyme system exhibited a substantial increase in the synthesis rate of methanol, which was about 4.5 times higher than that of the free enzyme system. Förster resonance energy transfer (FRET) analysis demonstrated that the tight arrangement of enzymes within MCF pores shortened the substrate transport pathway, thereby improving the catalytic activity [132].

**Figure 8:**
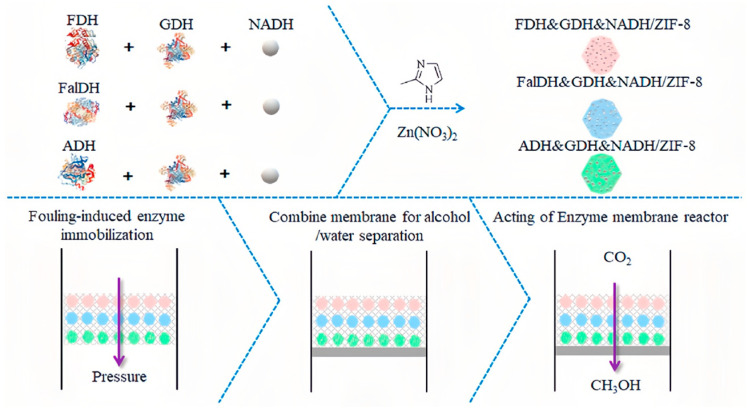
Ordered co-immobilization of the multi-enzyme cascade system with a metal–organic framework in a membrane: reduction of CO_2_ to methanol (see the Materials and Methods) [91].

According to the CO_2_ reduction process, Zhu et al. employed the dead-end filtering technique to layer nanocomposites containing FDH, GDH(Glu), and NADH in an ordered manner within the membrane (Figure 8). Methanol was effectively isolated from the alcohol–water mixture using permeable membrane technology. The enzyme reactor had the ability to accurately regulate the direction of the reaction, thus significantly improving the catalytic efficiency. The results indicated that the organized positioning of enzymes in the membrane could greatly enhance the effectiveness of the reaction, compared to the random positioning of enzymes. The methanol yield increased from 6.7 ± 0.8 μmol with a disordered arrangement to 12.6 ± 0.6 μmol with an ordered arrangement after 5 h of reaction. However, the limited conversion capacity of GDH(Glu) constrained the yield of methanol [91]. Liu et al. developed a multi-enzyme reactor, nFDH@ZIF-90@CA, using the positional co-immobilization method. By employing MV^2+^ as an electronic mediator, NADH could be effectively regenerated through electrochemical techniques, which provided a novel approach to enhancing the efficiency of CO_2_ conversion [82].

Position co-immobilization of multi-enzymes has been proven to be an efficient immobilization technique. An inherent benefit of this technology is its ability to independently optimize the microenvironment of each enzyme inside the system [135]. During the reaction, while the obstacle to mass transfer of intermediate products remains, the aforementioned issues can be mitigated by elevating the CO_2_ pressure to facilitate mass transfer. This approach has the potential to significantly enhance the efficiency of the substrate route.

Overall, improving the efficiency of multi-enzyme catalytic reactions relies on employing effective immobilization strategies. These tactics must conform to the following requirements: (1) The procedure of immobilization is simplified in order to minimize the risk of enzyme degradation. (2) The carrier possesses a large specific surface area, in order to accommodate a sufficient number of enzymes and effectively prevent any leakage. (3) The carrier has a porous structure to promote efficient diffusion of substrates and intermediates while removing the products to reduce inhibition of the enzyme. In addition, the most efficient method of enhancing the catalytic efficiency of numerous enzymes is to optimize the substrate route by altering the proportion and position of enzymes. Currently, there are several advanced techniques for immobilizing multi-enzymes that have demonstrated significant potential in improving the catalytic performance of enzymes. However, the pursuit of novel immobilization techniques that are both cost-effective and easy to implement remains a crucial area of investigation in this field.

## 4. Conclusions and Perspectives

Owing to their exceptional porosity and tunable pore structures, metal–organic framework materials (MOFs) have opened up new research avenues in the field of enzyme immobilization, particularly in the efficient catalytic conversion of CO_2_. The MOF materials offer notable advantages in facilitating the transport and diffusion of CO_2_ and products, hence markedly improving the catalytic efficiency and selectivity of multi-enzyme cascade reactions.

Despite significant progress in utilizing MOF-immobilized enzymes for CO_2_ conversion, there are several challenges remaining in achieving industrial-scale applications.

First, it is essential to develop MOF supports suitable for large-scale production that possess macroporous structures, enabling efficient enzyme immobilization and substrate mass transfer. Constructing MOFs with hierarchical pore structures to accommodate enzymes of various sizes presents a highly promising approach.

Second, functional modifications to MOF supports should be explored to improve enzyme stability and operational feasibility. This involves leveraging advances in synthetic chemistry and materials science to design MOFs with specific functionalities, such as smart MOFs responsive to environmental changes, or bio-based MOFs with enhanced biocompatibility and stability, thereby improving the activity and stability of immobilized enzymes.

Third, the development of novel MOFs with catalytic activity toward CO_2_ is a crucial strategy. On the one hand, the concept of machine learning can be utilized to explore the relationship between the enzyme structure and properties, aiming to design and screen novel MOF materials with high catalytic activity [136]. On the other hand, the construction of MOFs with photo- or electrocatalytic properties can significantly improve catalytic efficiency, and coupling these MOFs with other enzymes may enable the efficient conversion of CO_2_ into high-value carbon compounds.

In addition, maximizing the potential of multi-enzyme cascade systems to increase the yield remains a key research direction in the transformation of CO_2_ into multi-carbon compounds such as C2 and C4. Fully utilizing the structural diversity of MOFs to optimize substrate channeling effects in multi-enzyme cascade reactions is an effective strategy to improve catalytic selectivity. Furthermore, integrating computational biology and molecular dynamics simulations to predict and optimize enzyme–MOF interactions will help in constructing more efficient and specific multi-enzyme cascade catalytic systems. Simultaneously, understanding the interaction mechanisms between enzymes and MOFs requires further clarification, as it limits the optimization of immobilized enzyme system design. Future research should delve into the immobilization process of enzymes within MOFs, including how enzyme active sites interact with the MOF framework and how material design can enhance the stability and reusability of immobilized enzymes.

In conclusion, the application of MOF-immobilized enzyme technology in CO_2_ catalytic conversion holds great potential. However, further research and technical innovation are still required in the areas of material design, comprehension of interaction mechanisms, and multi-enzyme immobilization optimization. As these challenges are addressed, MOFs as enzyme carriers are anticipated to have a growing significance in upcoming biocatalysis procedures, and to offer a robust platform for the production of high-value chemicals from CO_2_.

## Figures and Tables

**Table 1:** Current applications of various coenzyme regeneration strategies in CO_2_ reduction processes using enzyme@MOFs.

NADH Regeneration System	Enzyme	Catalyst	Carriers	Product	Productivity	Reference
Chemical regeneration	/	Rh complex	/	NADH	0.2 mM·h^−1^	[89]
Enzymatic regeneration	CA, FDH, GDH	GDH	PEI@ZIF-8	Formate	13.8 mM·h^−1^	[90]
	FDH, FaldDH, ADH, GDH	GDH	ZIF-8@PVDF	Methanol	2.383 μmol·h^−1^	[91]
	CA, FDH, GDH	GDH	HKUST-1	Formate	0.87 mM·h^−1^	[74]
Electrochemical regeneration	FDH	NR	ZIF-8@SNF	Formate	0.46 mM·h^−1^	[59]
	FDH	NR	PEI@SBA-15	Formate	0.373 mM·h^−1^	[33]
	FDH	Rh complex	NU-1006	Formate	79.3 mM·h^−1^	[92]
	FDH, FaldDH, ADH	Rh complex	ZIF-8	Formate	0.107 mM·h^−1^	[93]
	FDH	MV	HP-UiO-66-NH_2_	Formate	0.609 mM·h^−1^	[40]
	CA, FDH	MV	ZIF-90	Formate	0.972 mM·h^−1^	[82]
Photochemical regeneration	FDH	Rh complex	H_2_TCPP-UiO-66-NH_2_	Formate	0.254 mM·h^−1^	[94]
	FDH, FaldDH	TCPP	TCPP-ZIF-8	Formaldehyde	0.968 mM·h^−1^	[95]
	FDH	Rh complex	NU-1006	Formate	79.34 mM·h^−1^	[92]
	FDH	CNMV	NU-1006	Formate	1.2 mM·h^−1^	[96]
	FDH	/	CdS-ZIF-67	Formate	0.51 mM·h^−1^	[97]
	FDH	Rh complex	In-CdS@ZIF-8	Formate	11.132 μM·h^−1^	[98]
	FDH	TPE-C_3_N_4_/PEI/Rh	MAF-7	Formate	1.861 mM·h^−1^	[99]
	CA, FDH	g-C_3_N_4_	ZIF-8	Formate	39.6 μM·h^−1^	[100]

**Table 2:** Overview of the multi-enzyme co-immobilization strategies employed for the catalytic conversion of CO_2_.

Co-Fixing Method	Immobilization Strategy	Enzyme	Carriers	Product *	Productivity	Reference
Random co-immobilization	In situ encapsulation	CA, FDH, GDH	PEI@ZIF-8	Formate	13.8 mM·h^−1^	[90]
	In situ encapsulation	FDH, FaldDH, ADH	ZIF-8	Methanol	0.107 mM·h^−1^	[93]
	In situ encapsulation	CA, FDH	PDA/PEI @ZIF-8	Formate	1.4 μmol·h^−1^	[130]
	In situ encapsulation	CA, FDH	PDA/PEI @UiO-66-NH_2_	Formate	0.925 μmol·h^−1^	[130]
	In situ encapsulation	CA, FDH	ZIF-8@g-C_3_N_4_	Formate	39.6 μM·h^−1^	[100]
	In situ encapsulation	CA, FDH	PDA/PEI @ZIF-8	Formate	8.13 mM·h^−1^	[131]
	In situ encapsulation	FDH, FaldDH	TCPP-ZIF-8	Formaldehyde	0.968 mM·h^−1^	[95]
Compartmental co-immobilization	In situ encapsulation	CA, FDH, GDH	HKUST-1	Formate	0.653 mM·h^−1^	[72]
Positional co-immobilization	In situ encapsulation	FDH, FaldDH, ADH, GDH	ZIF-8@PVDF	Methanol	2.383 μmol·h^−1^	[91]
	Surface fixation	FDH, ADH, FaldDH	MCF-MP	Methanol	1.35 mM·h^−1^	[132]
	In situ encapsulation Covalent linkage	CA, FDH	ZIF-90	Formate	0.972 mM·h^−1^	[82]

* CO_2_ is continuously supplied both before and during the reaction, maintaining a saturated concentration of CO_2_ throughout the reaction system.
